# Supplementary material for: Global disruption of coral broadcast spawning associated with artificial light at night
Source: Nat Commun. 2023 May 15;14:2511. doi: 10.1038/s41467-023-38070-y (PMC10185496; doi:10.1038/s41467-023-38070-y)
Supplement: Supplementary file 1 — Supplementary Information [file 41467_2023_38070_MOESM1_ESM.pdf]

Supplementary Information for Davies *et al*. 2023 "*Global disruption of coral broadcast spawning associated with artificial light at night*"

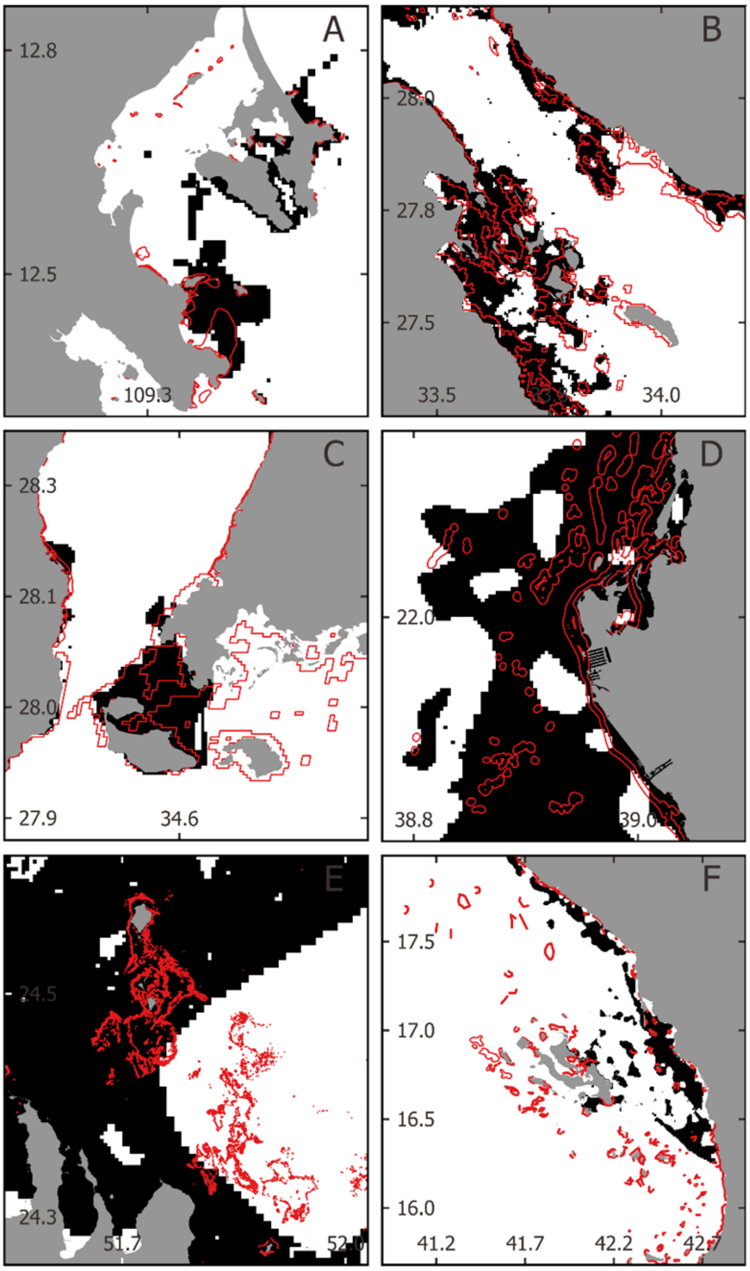

Supplementary Figure 1| Fine scale spatial heterogeneity in coral reef exposure to ALAN. Exposure to ALAN is indicated by black regions and determined by whether the depth of biologically relevant light pollution exceeds the depths at which reefs are located. Bathymetry was taken from GEBCO 2022 grid (doi:10.5285/e0f0bb80-ab44-2739-e053-6c86abc0289c). Reef locations (outlined in red) are taken from the UNEP-WCMC Global distribution of warm water coral reefs (2010) http://data.unep-wcmc.org/datasets/1. Numbers refer to latitude and longitude. Examples are given for: Ninh Diêm, Vietnam (**A**); the Giftun Islands and Straits of Gubal, Red Sea (**B**); Straits of Tiran, Red Sea (**C**); Dahaban, Saudi Arabia, Red Sea (**D**); Al Yasaf Marine Protect Area, Persian Gulf (**E**); and the Farasan Islands, Red Sea (**F**).

Supplementary Table 1| Model selection performed on all nested versions of a global generalised linear mixed effects model to explain the impact of: underwater light pollution (ALAN: 'Lit' or 'Unlit'); Genus; Distance from the Equator (DfE, degrees); the rate changes in annual average Sea Surface Temperature (ΔSST) and water clarity (ΔKd_{490}); and Ecoregion on the Day of Spawning Relative to the Nearest Full Moon (DoSRtNF). Terms included in the underlined models were included in the selected model for further interrogation. All models were fitted with a random effects term to account for spatial autocorrelation specified using the Matérn structure of pairwise correlations between the coordinates of each spawning location [+ Matern(1|longitude+latitude)].

**Model**	**AIC**	**weight**
<u>*DoSRtNF~ALAN*Genus + ALAN*DfE + Ecoregion + ΔSST*</u>	<u>9079.63</u>	<u>0.15</u>
<u>*DoSRtNF~ALAN*Genus + Ecoregion + ΔSST + DfE*</u>	<u>9080.69</u>	<u>0.09</u>
<u>*DoSRtNF~ALAN*Genus + ALAN*DfE + Ecoregion + ΔSST +* ΔKd_{490}</u>	<u>9081.21</u>	<u>0.07</u>
<u>*DoSRtNF~ALAN*Genus + ALAN*ΔSST + ALAN*DfE + Ecoregion*</u>	<u>9081.57</u>	<u>0.06</u>
*DoSRtNF~ALAN*Genus + ALAN* ΔSST + DfE + Ecoregion*	9082.13	0.04
*DoSRtNF~ALAN*Genus + ALAN* DfE+ Ecoregion*	9082.20	0.04
*DoSRtNF~ALAN*Genus + ALAN**ΔKd_{490} *+ ALAN*DfE + Ecoregion+ ΔSST*	9082.2	0.04
*DoSRtNF~ALAN*Genus + ALAN**ΔKd_{490}*+ Ecoregion + ΔSST + DfE*	9082.49	0.04
*DoSRtNF~ ALAN*Genus +* ΔKd_{490} *+ Ecoregion + ΔSST + DfE*	9082.54	0.04
*DoSRtNF~ALAN*Genus + ALAN*DfE + Ecoregion +* ΔKd_{490}	9082.54	0.04
*DoSRtNF~ALAN*Genus + Ecoregion + DfE*	9082.61	0.03
*DoSRtNF~ALAN*Genus + ALAN*ΔSST + ALAN*DfE + Ecoregion +* ΔKd_{490}	9083.12	0.03
*DoSRtNF~ALAN*Genus + ALAN*ΔSST + ALAN**ΔKd_{490} *+ Ecoregion + DfE*	9083.26	0.02
*DoSRtNF~ALAN*Genus + Ecoregion +* ΔKd_{490} *+ DfE*	9083.58	0.02
*DoSRtNF~ALAN*Genus + Ecoregion +* ΔKd_{490}	9083.63	0.02
*DoSRtNF~ALAN*Genus + ALAN**ΔKd_{490} *+ ALAN*DfE+ Ecoregion*	9083.73	0.02
*DoSRtNF~ALAN*Genus + ALAN**ΔKd_{490} *+ ALAN*DfE + ALAN*ΔSST +Ecoregion*	9083.79	0.02
*DoSRtNF~ALAN*Genus +* ΔKd_{490}	9083.80	0.02
*DoSRtNF~ALAN*Genus + ALAN**ΔKd_{490} *+ Ecoregion + DfE*	9083.3	0.02
*DoSRtNF~ALAN*Genus + ALAN* ΔSST +* ΔKd_{490} *+ DfE + Ecoregion*	9083.88	0.02

Supplementary Table 2| Pairwise comparisons of the Day of Spawning Relative to the Nearest Full Moon (DoSRtNF) for observations recorded for each Genus from locations exposed (Lit) and not exposed (Unlit) to underwater light pollution. Comparisons are derived from spatially autocorrelated mixed effects models fitted independently for each genus. Results are presented in Figure 2A. P values were not corrected for multiple tests.

Genus	Unlit-Lit	SE	*Z*	*p*	*n* Lit	*n* Unlit
Acanthastrea spp.[a]	0.30	0.15	1.98	0.048	22	10
Acropora spp.[b]	0.70	0.25	2.86	0.004	208	575
Cyphastrea spp.[a]	0.54	0.20	2.77	0.006	22	17
Dipsastraea spp.[a]	0.48	0.22	2.23	0.026	25	50
Echinophyllia spp.[a]	0.26	0.14	1.81	0.070	23	21
Favites spp.[a]	0.28	0.12	2.33	0.02	273	92
Galaxea spp.[b]	0.41	0.15	2.64	0.008	72	106
Goniastrea spp.[a]	0.36	0.10	3.83	<0.001	83	27
Leptoria spp.[a]	0.14	0.23	0.61	0.543	6	8
Montipora spp.[a]	0.48	0.22	2.16	0.031	60	69
Platygyra spp.[b]	0.29	0.12	2.49	0.012	115	165
Porites spp.[a]	0.80	0.12	6.91	<0.001	59	27

[a]Models fitted using poisson error distribution for dispersed data
[b]Models fitted using negative binomial error distribution for overdispersed data

Supplementary Table 3| Predicted model means and confidence intervals derived from spatially autocorrelated mixed effects models fitted independently to each genus. Means and confidence intervals are presented on the scale of the response here and in Figure 2A. Random effects modelled due to spatial autocorrelation were removed from the prediction of means and confidence intervals.

Genus	ALAN	Predicted DoSRtNFM	lower 95% CI	upper 95% CI
Acanthastrea spp.[a]	Lit	5.18	4.31	6.23
	Unlit	7.00	5.54	8.85
Acropora spp.[b]	Lit	2.27	1.52	3.40
	Unlit	4.58	3.44	6.08
Cyphastrea spp.[a]	Lit	2.76	1.61	4.71
	Unlit	4.73	2.91	7.68
Dipsastraea spp.[a]	Lit	3.44	2.46	4.81
	Unlit	5.57	4.30	7.21
Echinophyllia spp.[a]	Lit	4.83	3.96	5.88
	Unlit	6.23	5.14	7.56
Favites spp.[a]	Lit	4.41	3.67	5.30
	Unlit	5.81	4.79	7.06
Galaxea spp.[b]	Lit	4.36	3.45	5.51
	Unlit	6.54	5.41	7.91
Goniastrea spp.[a]	Lit	4.17	3.75	4.63
	Unlit	6.00	5.14	7.00
Leptoria spp.[a]	Lit	5.33	3.77	7.54
	Unlit	6.13	4.63	8.10
Montipora spp.[a]	Lit	2.43	1.63	3.61
	Unlit	3.91	2.78	5.51
Platygyra spp.[b]	Lit	5.04	4.19	6.07
	Unlit	6.74	5.74	7.91
Porites spp.[a]	Lit	2.49	2.12	2.93
	Unlit	5.56	4.73	6.52

[a]Models fitted using poisson error distribution for dispersed data
[b]Models fitted using negative binomial error distribution for overdispersed data

Supplementary Table 4| The distribution of coral spawning observations across species.

Species	*n* observations	Species	*n* observations
Acanthastrea brevis	4	*Acropora papillare*	5
Acanthastrea echinata	26	*Acropora parapharaonis*	2
Acanthastrea rotundoflora	2	*Acropora pharaonis*	5
Acropora abrotanoides	1	*Acropora plantaginea*	8
Acropora acuminata	2	*Acropora pocilloporina*	1
Acropora akajimensis	6	*Acropora polystoma*	2
Acropora anthocercis	12	*Acropora pulchra*	14
Acropora arabensis	1	*Acropora retusa*	4
Acropora arafura	1	*Acropora robusta*	9
Acropora aspera	2	*Acropora samoensis*	5
Acropora austera	2	*Acropora sarmentosa*	15
Acropora carduus	3	*Acropora secale*	6
Acropora cerealis	3	*Acropora selago*	10
Acropora clathrata	3	*Acropora solitaryensis*	19
Acropora copiosa	1	*Acropora sp 1 Fiji*	7
Acropora cytherea	19	*Acropora sp 2 Fiji*	3
Acropora dendrum	4	*Acropora sp 3 Fiji*	2
Acropora digitifera	21	*Acropora sp 4 Fiji*	3
Acropora divaricata	2	*Acropora sp 7 Fiji*	2
Acropora downingi	2	*Acropora sp 8 Fiji*	1
Acropora elseyi	1	*Acropora sp 9 Fiji*	2
Acropora eurystoma	14	*Acropora sp.*	2
Acropora florida	23	*Acropora spathulata*	1
Acropora gemmifera	47	*Acropora spicifera*	2
Acropora globiceps	1	*Acropora squarrosa*	3
Acropora hemprichii	4	*Acropora striata*	3
Acropora humilis	36	*Acropora tenuis*	57
Acropora hyacinthus	96	*Acropora tumida*	6
Acropora insignis	3	*Acropora valida*	20
Acropora intermedia	18	*Acropora variolosa*	5
Acropora japonica	12	*Acropora vaughani*	14
Acropora lamarcki	3	*Acropora verweyi*	11
Acropora latistella	2	*Acropora willisae*	4
Acropora loripes	10	*Acropora yongei*	3
Acropora lutkeni	9	*Cyphastrea chalcidicum*	18
Acropora maryae	1	*Cyphastrea japonica*	2
Acropora microclados	4	*Cyphastrea microphthalma*	10
Acropora microphthalma	6	*Cyphastrea serailia*	9
Acropora millepora	51	*Dipsastraea danai*	1
Acropora monticulosa	4	*Dipsastraea faviaformis*	2
Acropora muricata	46	*Dipsastraea favus*	20
Acropora nana	4	*Dipsastraea lizardensis*	4
Acropora nasuta	46	*Dipsastraea matthaii*	4
Acropora ocellata	1	*Dipsastraea pallida*	12

Species	*n* observations	Species	*n* observations
Dipsastraea sp.	1	*Montipora efflorescens*	6
Dipsastraea speciosa	28	*Montipora hispida*	20
Dipsastraea truncata	2	*Montipora hoffmeisteri*	1
Dipsastraea veroni	1	*Montipora informis*	20
Echinophyllia aspera	29	*Montipora malampaya*	4
Echinophyllia echinoporoides	14	*Montipora samarensis*	2
Echinophyllia orpheensis	1	*Montipora sp 1 Fiji*	2
Favites abdita	52	*Montipora sp.*	5
Favites acuticollis	2	*Montipora spongodes*	3
Favites chinensis	10	*Montipora stellata*	9
Favites colemani	3	*Montipora tuberculosa*	3
Favites complanata	15	*Montipora turgescens*	9
Favites flexuosa	5	*Montipora venosa*	18
Favites halicora	2	*Montipora verrucosa*	3
Favites magnistellata	16	*Platygyra contorta*	29
Favites paraflexuosus	1	*Platygyra daedalea*	30
Favites pentagona	242	*Platygyra lamellina*	108
Favites sp.	1	*Platygyra pini*	23
Favites spinosa	1	*Platygyra ryukyuensis*	22
Favites stylifera	2	*Platygyra sinensis*	63
Favites valenciennesi	13	*Platygyra sp.*	1
Galaxea astreata	36	*Platygyra verweyi*	4
Galaxea fascicularis	142	*Porites *massive*	23
Goniastrea edwardsi	16	*Porites columnaris*	2
Goniastrea favulus	4	*Porites cylindrica*	2
Goniastrea minuta	20	*Porites lobata*	2
Goniastrea pectinata	39	*Porites lutea*	54
Goniastrea retiformis	23	*Porites rus*	1
Goniastrea stelligera	8	*Porites solida*	2
Leptoria phrygia	14		
Montipora aequituberculata	5		
Montipora altasepta	2		
Montipora cactus	3		
Montipora caliculata	1		
Montipora cocosensis	1		
Montipora corbettensis	8		
Montipora crassituberculata	2		
Montipora danae	1		
Montipora digitata	1		

Supplementary Table 5| Pairwise comparisons of the Day of Spawning Relative to the Nearest Full Moon (DoSRtNF) for observations recorded from different coral ecoregions around the world. Comparisons are derived from negative binomial spatially autocorrelated mixed effects models fitted independently for ecoregion. Results are presented in Figure 2C. P values were not corrected for multiple tests.

Contrast	Estimate	*SE*	*t*	*p*
Bismarck Sea, Papua New Guinea - Central and northern Great Barrier Reef	-0.14	0.46	-0.3	0.767
Bismarck Sea, Papua New Guinea - Fiji	0.24	0.45	0.53	0.594
Bismarck Sea, Papua New Guinea - Gulf of Thailand	0.08	0.42	0.2	0.845
Bismarck Sea, Papua New Guinea - Mascarene Islands	-0.08	0.46	-0.17	0.865
Bismarck Sea, Papua New Guinea - North and central Red Sea	-0.4	0.4	-1.01	0.314
Bismarck Sea, Papua New Guinea - North Philippines	0.38	0.45	0.85	0.397
Bismarck Sea, Papua New Guinea - North Ryukyu Islands, Japan	-0.47	0.43	-1.09	0.274
Bismarck Sea, Papua New Guinea - Palau	-0.26	0.48	-0.55	0.585
Bismarck Sea, Papua New Guinea - Scott Reef, west Australia	0.57	0.51	1.11	0.269
Bismarck Sea, Papua New Guinea - Shikoku, Japan	0.53	0.45	1.18	0.239
Bismarck Sea, Papua New Guinea - Society Islands, French Polynesia	0.52	0.51	1.01	0.311
<u>Bismarck Sea, Papua New Guinea - South Ryukyu Islands, Japan</u>	<u>-1.2</u>	<u>0.44</u>	<u>-2.75</u>	<u>0.006</u>
Bismarck Sea, Papua New Guinea - South-east Kyushu, Japan	0.56	0.51	1.1	0.272
Bismarck Sea, Papua New Guinea - Strait of Malacca, eastern Sumatra and Singapore	-0.02	0.51	-0.05	0.962
Bismarck Sea, Papua New Guinea - Sulu Sea, Philippines	-0.36	0.45	-0.79	0.429
<u>Bismarck Sea, Papua New Guinea - Sunda Shelf, south-east Asia</u>	<u>-1.07</u>	<u>0.48</u>	<u>-2.2</u>	<u>0.028</u>
Bismarck Sea, Papua New Guinea - Taiwan and coastal China	0.19	0.4	0.46	0.644
Bismarck Sea, Papua New Guinea - West and south Madagascar	0.51	0.52	0.97	0.331
Central and northern Great Barrier Reef - Fiji	0.38	0.36	1.03	0.301
Central and northern Great Barrier Reef - Gulf of Thailand	0.22	0.33	0.66	0.511
Central and northern Great Barrier Reef - Mascarene Islands	0.06	0.38	0.16	0.877
Central and northern Great Barrier Reef - North and central Red Sea	-0.26	0.3	-0.88	0.379
Central and northern Great Barrier Reef - North Philippines	0.52	0.37	1.42	0.156
Central and northern Great Barrier Reef - North Ryukyu Islands, Japan	-0.33	0.34	-0.98	0.328
Central and northern Great Barrier Reef - Palau	-0.13	0.41	-0.31	0.754
Central and northern Great Barrier Reef - Scott Reef, west Australia	0.71	0.44	1.59	0.111
Central and northern Great Barrier Reef - Shikoku, Japan	0.67	0.36	1.83	0.068
Central and northern Great Barrier Reef - Society Islands, French Polynesia	0.66	0.44	1.49	0.137
<u>Central and northern Great Barrier Reef - South Ryukyu Islands, Japan</u>	<u>-1.06</u>	<u>0.35</u>	<u>-3.05</u>	<u>0.002</u>
Central and northern Great Barrier Reef - South-east Kyushu, Japan	0.7	0.44	1.59	0.112
Central and northern Great Barrier Reef - Strait of Malacca, eastern Sumatra and Singapore	0.11	0.43	0.26	0.796
Central and northern Great Barrier Reef - Sulu Sea, Philippines	-0.22	0.37	-0.6	0.548
<u>Central and northern Great Barrier Reef - Sunda Shelf, south-east Asia</u>	<u>-0.93</u>	<u>0.41</u>	<u>-2.28</u>	<u>0.023</u>
Central and northern Great Barrier Reef - Taiwan and coastal China	0.32	0.31	1.06	0.29
Central and northern Great Barrier Reef - West and south Madagascar	0.65	0.45	1.42	0.155

Fiji - Gulf of Thailand	-0.16	0.32	-0.49	0.623
Fiji - Mascarene Islands	-0.32	0.36	-0.87	0.382
Fiji - North and central Red Sea	-0.64	0.28	-2.28	0.023
Fiji - North Philippines	0.14	0.35	0.41	0.683
Fiji - North Ryukyu Islands, Japan	-0.7	0.32	-2.21	0.027
Fiji - Palau	-0.5	0.39	-1.28	0.201
Fiji - Scott Reef, west Australia	0.33	0.43	0.77	0.442
Fiji - Shikoku, Japan	0.29	0.35	0.83	0.404
Fiji - Society Islands, French Polynesia	0.28	0.43	0.66	0.512
Fiji - South Ryukyu Islands, Japan	-1.44	0.33	-4.34	0
Fiji - South-east Kyushu, Japan	0.32	0.43	0.76	0.448
Fiji - Strait of Malacca, eastern Sumatra and Singapore	-0.26	0.42	-0.63	0.532
Fiji - Sulu Sea, Philippines	-0.6	0.35	-1.69	0.091
Fiji - Sunda Shelf, south-east Asia	-1.3	0.39	-3.32	0.001
Fiji - Taiwan and coastal China	-0.05	0.29	-0.18	0.854
Fiji - West and south Madagascar	0.27	0.44	0.62	0.538
Gulf of Thailand - Mascarene Islands	-0.16	0.33	-0.48	0.63
Gulf of Thailand - North and central Red Sea	-0.48	0.24	-1.99	0.046
Gulf of Thailand - North Philippines	0.3	0.32	0.93	0.35
Gulf of Thailand - North Ryukyu Islands, Japan	-0.55	0.29	-1.92	0.055
Gulf of Thailand - Palau	-0.35	0.37	-0.95	0.344
Gulf of Thailand - Scott Reef, west Australia	0.49	0.41	1.2	0.231
Gulf of Thailand - Shikoku, Japan	0.45	0.32	1.4	0.161
Gulf of Thailand - Society Islands, French Polynesia	0.44	0.4	1.08	0.28
Gulf of Thailand - South Ryukyu Islands, Japan	-1.28	0.3	-4.28	0
Gulf of Thailand - South-east Kyushu, Japan	0.48	0.4	1.19	0.233
Gulf of Thailand - Strait of Malacca, eastern Sumatra and Singapore	-0.11	0.4	-0.27	0.787
Gulf of Thailand - Sulu Sea, Philippines	-0.44	0.32	-1.36	0.173
Gulf of Thailand - Sunda Shelf, south-east Asia	-1.15	0.37	-3.13	0.002
Gulf of Thailand - Taiwan and coastal China	0.1	0.25	0.42	0.678
Gulf of Thailand - West and south Madagascar	0.43	0.42	1.02	0.307
Mascarene Islands - North and central Red Sea	-0.32	0.3	-1.08	0.281
Mascarene Islands - North Philippines	0.46	0.36	1.26	0.208
Mascarene Islands - North Ryukyu Islands, Japan	-0.39	0.34	-1.16	0.248
Mascarene Islands - Palau	-0.19	0.41	-0.46	0.647
Mascarene Islands - Scott Reef, west Australia	0.65	0.44	1.46	0.144
Mascarene Islands - Shikoku, Japan	0.61	0.36	1.67	0.095
Mascarene Islands - Society Islands, French Polynesia	0.6	0.44	1.36	0.175
Mascarene Islands - South Ryukyu Islands, Japan	-1.12	0.35	-3.22	0.001
Mascarene Islands - South-east Kyushu, Japan	0.64	0.44	1.46	0.144
Mascarene Islands - Strait of Malacca, eastern Sumatra and Singapore	0.05	0.43	0.12	0.901
Mascarene Islands - Sulu Sea, Philippines	-0.28	0.37	-0.76	0.447
Mascarene Islands - Sunda Shelf, south-east Asia	-0.99	0.41	-2.43	0.015
Mascarene Islands - Taiwan and coastal China	0.26	0.3	0.87	0.386
Mascarene Islands - West and south Madagascar	0.59	0.45	1.3	0.195

North and central Red Sea - North Philippines	0.78	0.28	2.76	0.006
North and central Red Sea - North Ryukyu Islands, Japan	-0.06	0.25	-0.26	0.793
North and central Red Sea - Palau	0.14	0.34	0.41	0.683
North and central Red Sea - Scott Reef, west Australia	0.97	0.38	2.56	0.01
North and central Red Sea - Shikoku, Japan	0.93	0.28	3.29	0.001
North and central Red Sea - Society Islands, French Polynesia	0.92	0.38	2.45	0.014
North and central Red Sea - South Ryukyu Islands, Japan	-0.8	0.26	-3.05	0.002
North and central Red Sea - South-east Kyushu, Japan	0.96	0.37	2.58	0.01
North and central Red Sea - Strait of Malacca, eastern Sumatra and Singapore	0.38	0.37	1.02	0.305
North and central Red Sea - Sulu Sea, Philippines	0.04	0.29	0.15	0.883
North and central Red Sea - Sunda Shelf, south-east Asia	-0.66	0.34	-1.97	0.049
North and central Red Sea - Taiwan and coastal China	0.59	0.2	2.92	0.003
North and central Red Sea - West and south Madagascar	0.91	0.39	2.33	0.02
North Philippines - North Ryukyu Islands, Japan	-0.85	0.32	-2.64	0.008
North Philippines - Palau	-0.64	0.39	-1.63	0.102
North Philippines - Scott Reef, west Australia	0.19	0.43	0.44	0.663
North Philippines - Shikoku, Japan	0.15	0.35	0.42	0.673
North Philippines - Society Islands, French Polynesia	0.14	0.43	0.32	0.748
North Philippines - South Ryukyu Islands, Japan	-1.58	0.33	-4.74	0
North Philippines - South-east Kyushu, Japan	0.18	0.43	0.42	0.673
North Philippines - Strait of Malacca, eastern Sumatra and Singapore	-0.41	0.42	-0.96	0.337
North Philippines - Sulu Sea, Philippines	-0.74	0.36	-2.08	0.038
North Philippines - Sunda Shelf, south-east Asia	-1.45	0.4	-3.66	0
North Philippines - Taiwan and coastal China	-0.19	0.29	-0.68	0.499
North Philippines - West and south Madagascar	0.13	0.44	0.29	0.771
North Ryukyu Islands, Japan - Palau	0.2	0.37	0.55	0.583
North Ryukyu Islands, Japan - Scott Reef, west Australia	1.04	0.41	2.54	0.011
North Ryukyu Islands, Japan - Shikoku, Japan	1	0.32	3.1	0.002
North Ryukyu Islands, Japan - Society Islands, French Polynesia	0.99	0.41	2.43	0.015
North Ryukyu Islands, Japan - South Ryukyu Islands, Japan	-0.73	0.3	-2.42	0.015
North Ryukyu Islands, Japan - South-east Kyushu, Japan	1.03	0.4	2.55	0.011
North Ryukyu Islands, Japan - Strait of Malacca, eastern Sumatra and Singapore	0.44	0.4	1.11	0.267
North Ryukyu Islands, Japan - Sulu Sea, Philippines	0.11	0.33	0.33	0.742
North Ryukyu Islands, Japan - Sunda Shelf, south-east Asia	-0.6	0.37	-1.62	0.104
North Ryukyu Islands, Japan - Taiwan and coastal China	0.65	0.25	2.59	0.009
North Ryukyu Islands, Japan - West and south Madagascar	0.98	0.42	2.32	0.02
Palau - Scott Reef, west Australia	0.83	0.47	1.78	0.075
Palau - Shikoku, Japan	0.79	0.39	2.01	0.044
Palau - Society Islands, French Polynesia	0.78	0.47	1.68	0.093
Palau - South Ryukyu Islands, Japan	-0.93	0.38	-2.46	0.014
Palau - South-east Kyushu, Japan	0.83	0.46	1.78	0.075
Palau - Strait of Malacca, eastern Sumatra and Singapore	0.24	0.46	0.52	0.601
Palau - Sulu Sea, Philippines	-0.09	0.4	-0.24	0.812
Palau - Sunda Shelf, south-east Asia	-0.8	0.43	-1.85	0.065
Palau - Taiwan and coastal China	0.45	0.34	1.32	0.186

Palau - West and south Madagascar	0.77	0.48	1.62	0.106
Scott Reef, west Australia - Shikoku, Japan	-0.04	0.43	-0.09	0.926
Scott Reef, west Australia - Society Islands, French Polynesia	-0.05	0.5	-0.1	0.919
Scott Reef, west Australia - South Ryukyu Islands, Japan	-1.77	0.42	-4.23	0
Scott Reef, west Australia - South-east Kyushu, Japan	-0.01	0.5	-0.02	0.987
Scott Reef, west Australia - Strait of Malacca, eastern Sumatra and Singapore	-0.59	0.49	-1.21	0.227
Scott Reef, west Australia - Sulu Sea, Philippines	-0.93	0.44	-2.13	0.033
Scott Reef, west Australia - Sunda Shelf, south-east Asia	-1.64	0.47	-3.49	0
Scott Reef, west Australia - Taiwan and coastal China	-0.38	0.38	-1	0.316
Scott Reef, west Australia - West and south Madagascar	-0.06	0.51	-0.12	0.908
Shikoku, Japan - Society Islands, French Polynesia	-0.01	0.43	-0.02	0.981
Shikoku, Japan - South Ryukyu Islands, Japan	-1.73	0.33	-5.2	0
Shikoku, Japan - South-east Kyushu, Japan	0.03	0.43	0.08	0.94
Shikoku, Japan - Strait of Malacca, eastern Sumatra and Singapore	-0.55	0.42	-1.31	0.189
Shikoku, Japan - Sulu Sea, Philippines	-0.89	0.36	-2.5	0.012
Shikoku, Japan - Sunda Shelf, south-east Asia	-1.6	0.39	-4.04	0
Shikoku, Japan - Taiwan and coastal China	-0.34	0.29	-1.19	0.233
Shikoku, Japan - West and south Madagascar	-0.02	0.44	-0.04	0.966
Society Islands, French Polynesia - South Ryukyu Islands, Japan	-1.72	0.41	-4.14	0
Society Islands, French Polynesia - South-east Kyushu, Japan	0.04	0.49	0.09	0.931
Society Islands, French Polynesia - Strait of Malacca, eastern Sumatra and Singapore	-0.54	0.49	-1.11	0.267
Society Islands, French Polynesia - Sulu Sea, Philippines	-0.88	0.43	-2.03	0.043
Society Islands, French Polynesia - Sunda Shelf, south-east Asia	-1.59	0.47	-3.4	0.001
Society Islands, French Polynesia - Taiwan and coastal China	-0.33	0.38	-0.88	0.38
Society Islands, French Polynesia - West and south Madagascar	-0.01	0.51	-0.02	0.986
South Ryukyu Islands, Japan - South-east Kyushu, Japan	1.76	0.41	4.26	0
South Ryukyu Islands, Japan - Strait of Malacca, eastern Sumatra and Singapore	1.17	0.41	2.88	0.004
South Ryukyu Islands, Japan - Sulu Sea, Philippines	0.84	0.34	2.48	0.013
South Ryukyu Islands, Japan - Sunda Shelf, south-east Asia	0.13	0.38	0.35	0.73
South Ryukyu Islands, Japan - Taiwan and coastal China	1.38	0.27	5.2	0
South Ryukyu Islands, Japan - West and south Madagascar	1.71	0.43	3.98	0
South-east Kyushu, Japan - Strait of Malacca, eastern Sumatra and Singapore	-0.59	0.49	-1.2	0.229
South-east Kyushu, Japan - Sulu Sea, Philippines	-0.92	0.43	-2.13	0.033
South-east Kyushu, Japan - Sunda Shelf, south-east Asia	-1.63	0.46	-3.51	0
South-east Kyushu, Japan - Taiwan and coastal China	-0.38	0.38	-1	0.32
South-east Kyushu, Japan - West and south Madagascar	-0.05	0.51	-0.1	0.919
Strait of Malacca, eastern Sumatra and Singapore - Sulu Sea, Philippines	-0.33	0.43	-0.78	0.433
Strait of Malacca, eastern Sumatra and Singapore - Sunda Shelf, south-east Asia	-1.04	0.46	-2.27	0.023
Strait of Malacca, eastern Sumatra and Singapore - Taiwan and coastal China	0.21	0.37	0.57	0.571
Strait of Malacca, eastern Sumatra and Singapore - West and south Madagascar	0.53	0.5	1.07	0.286
Sulu Sea, Philippines - Sunda Shelf, south-east Asia	-0.71	0.4	-1.77	0.077

Sulu Sea, Philippines - Taiwan and coastal China	0.54	0.29	1.85	0.064
Sulu Sea, Philippines - West and south Madagascar	0.87	0.45	1.94	0.052
Sunda Shelf, south-east Asia - Taiwan and coastal China	1.25	0.34	3.67	0
Sunda Shelf, south-east Asia - West and south Madagascar	1.58	0.48	3.29	0.001
Taiwan and coastal China - West and south Madagascar	0.32	0.4	0.82	0.412

Supplementary Table 6| Ecoregions included in the analysis. Ecoregion ID refers to the numerical identifiers given on the x-axis of Fig 2C. The number of lit and unlit observations within each Ecoregion are given.

Ecoregion ID	Ecoregion name	*n* lit	*n* unlit
78	Central and northern Great Barrier Reef	0	24
96	Scott Reef, west Australia	0	14
111	Fiji	0	235
18	Mascarene Islands	21	0
115	Society Islands, French Polynesia	0	20
1	North and central Red Sea	154	215
58	North Ryukyu Islands, Japan	127	23
61	Shikoku, Japan	0	182
57	South Ryukyu Islands, Japan	1	28
45	Sulu Sea, Philippines	0	47
49	Sunda Shelf, south-east Asia	18	0
66	Palau	1	28
73	Bismarck Sea, Papua New Guinea	0	9
47	North Philippines	1	62
32	Strait of Malacca, eastern Sumatra and Singapore	310	0
56	Taiwan and coastal China	314	243
50	Gulf of Thailand	21	4
17	West and south Madagascar	0	7
59	South-east Kyushu, Japan	0	26

Supplementary Table 7| Parameters used by the Gregg and Carder[27] model. Marine aerosol model utilized.

Parameter	Symbol	Value
Atmospheric pressure	p0	1013.25 mb
Ozone concentration	O_3	300 Dobson Units
Precipitable water vapour		1.5 cm
Relative humidity	RH	80%
Wind speed		5.0 ms^{-1}
Visibility		15.0 km
