# Supplementary material for: Global disruption of coral broadcast spawning associated with artificial light at night
Source: Nat Commun. 2023 May 15;14:2511. doi: 10.1038/s41467-023-38070-y (PMC10185496; doi:10.1038/s41467-023-38070-y)
Supplement: Supplementary file 3 — Reporting Summary [file 41467_2023_38070_MOESM3_ESM.pdf]

nature portfolio

Corresponding author(s): Thomas Davies

Last updated by author(s): 15/03/2023

Reporting Summary

Nature Portfolio wishes to improve the reproducibility of the work that we publish. This form provides structure for consistency and transparency in reporting. For further information on Nature Portfolio policies, see our Editorial Policies and the Editorial Policy Checklist.

Statistics

For all statistical analyses, confirm that the following items are present in the figure legend, table legend, main text, or Methods section.

n/a	Confirmed	
[ ]	[x]	The exact sample size (*n*) for each experimental group/condition, given as a discrete number and unit of measurement
[x]	[ ]	A statement on whether measurements were taken from distinct samples or whether the same sample was measured repeatedly
[ ]	[x]	The statistical test(s) used AND whether they are one- or two-sided<br>*Only common tests should be described solely by name; describe more complex techniques in the Methods section.*
[ ]	[x]	A description of all covariates tested
[ ]	[x]	A description of any assumptions or corrections, such as tests of normality and adjustment for multiple comparisons
[ ]	[x]	A full description of the statistical parameters including central tendency (e.g. means) or other basic estimates (e.g. regression coefficient) AND variation (e.g. standard deviation) or associated estimates of uncertainty (e.g. confidence intervals)
[ ]	[x]	For null hypothesis testing, the test statistic (e.g. F, t, r) with confidence intervals, effect sizes, degrees of freedom and P value noted<br>*Give P values as exact values whenever suitable.*
[x]	[ ]	For Bayesian analysis, information on the choice of priors and Markov chain Monte Carlo settings
[x]	[ ]	For hierarchical and complex designs, identification of the appropriate level for tests and full reporting of outcomes
[ ]	[x]	Estimates of effect sizes (e.g. Cohen's *d*, Pearson's *r*), indicating how they were calculated

Our web collection on statistics for biologists contains articles on many of the points above.

Software and code

Policy information about availability of computer code

Data collection	The code used to generate the light cycles in Fig 3 is available at https://github.com/timjsmyth/TidalLight<br>TidalLight uses python modules astropy (http://www.astropy.org/), pysolar (https://doi.org/10.5281/zenodo.1461066) and UTide (https://github.com/wesleybowman/UTide)
Data analysis	The data analysis was conducted in R v3.6.1

For manuscripts utilizing custom algorithms or software that are central to the research but not yet described in published literature, software must be made available to editors and reviewers. We strongly encourage code deposition in a community repository (e.g. GitHub). See the Nature Portfolio guidelines for submitting code & software for further information.

Data

Policy information about availability of data

All manuscripts must include a data availability statement. This statement should provide the following information, where applicable:

- Accession codes, unique identifiers, or web links for publicly available datasets
- A description of any restrictions on data availability
- For clinical datasets or third party data, please ensure that the statement adheres to our policy

The global atlas of artificial light at night under the sea is available to download from https://doi.pangaea.de/10.1594/PANGAEA.929749. The coral spawning database is available from https://doi.org/10.25405/data.ncl.13082333.v1. The data used in the analysis are provided in the Supplementary Information/Source

Data file.

Human research participants

Policy information about studies involving human research participants and Sex and Gender in Research.

Reporting on sex and gender	No human research participants were used in the study
Population characteristics	No human research participants were used in the study
Recruitment	No human research participants were used in the study
Ethics oversight	No human research participants were used in the study

Note that full information on the approval of the study protocol must also be provided in the manuscript.

Field-specific reporting

Please select the one below that is the best fit for your research. If you are not sure, read the appropriate sections before making your selection.

☐ Life sciences ☐ Behavioural & social sciences ☒ Ecological, evolutionary & environmental sciences

For a reference copy of the document with all sections, see nature.com/documents/nr-reporting-summary-flat.pdf

Ecological, evolutionary & environmental sciences study design

All studies must disclose on these points even when the disclosure is negative.

Study description	We analysed a dataset of 2135 coral broadcast spawning observations to quantify whether underwater ALAN disrupted the timing of broadcast spawning relative to the full moon. Our analysis accounted for: taxonomic differences (Genus, factor with 12 levels); the rate changes in average annual nighttime sea surface temperature (ΔSST, numerical) and water clarity (diffuse attenuation coefficient at 490nm, ΔKd490, numerical) over the year 2003 to 2022 period; latitudinal effects (Distance from the Equator, DfE, numerical); differences in spawning times between ecoregions (Ecoregion, factor with 19 levels); and spatial autocorrelation between sampling locations. Model selection was performed on all nested versions of a global spatially autocorrelated generalized linear mixed effects model [DoSRtNF~ALAN*Genus + ALAN*ΔSST + ALAN*ΔKd490 + ALAN*DfE + Ecoregion + Matern(1|Longitude+Latitude)] fitted with a poisson error distribution (dispersion = 0.82).
Research sample	We combined the recently published 'Global atlas of artificial light at night under the sea' (accessed from https://doi.pangaea.de/10.1594/PANGAEA.929749 on 15/11/2021) with the Coral Spawning Database (accessed from https://doi.org/10.25405/data.ncl.13082333.v1 on 25/11/2021), to establish whether broadcast spawning by scleractinian corals is disrupted in artificially lit waters. At the time it was accessed, the coral spawning database contained 6178 observations recorded at 101 locations around the world between 1978 and 2019, across 330 species representing 61 genera. Observations are recorded both as the calendar day, and the number of days relative to the nearest full moon (DoSRtNF). The analysis was restricted to spawning observations recorded in the 21st century such that the global atlas of artificial light at night under the sea is broadly representative of probable light exposure during the time of observations. Genera with less than six recorded observations per ALAN exposure level were excluded from the analysis. Data from ecoregions with fewer than six recorded observations were excluded from the analysis. Ex situ observations of coral spawning were omitted from the analysis to remove the influence of nearby light sources from buildings and laboratory light sources. The resulting dataset consisted of 2135 spawning observations recorded at 52 locations in 19 Ecoregions around the world between 2000 and 2019, across 156 species representing 12 genera.
Sampling strategy	The coral spawning database provided the key response variable for the analysis and the predictors 'Genus' and 'Distance from the Equator'. The 'global atlas of artificial light at night under the sea' provided the data for defining spawning obervations as 'lit' or 'unlit' ,a key predictor variable of interest. The MODIS AQUA Sea Surface Temperature and Kd490 datasets provided the time series data from which rate changes in STT and water clarity were quantified, two key predictor variables of interest.
Data collection	Data was collected using web searches for existing datasets over the relevant time range and of sufficient spatial resolution.
Timing and spatial scale	Data was accessed at the the point of need following commencement of the analysis.
Data exclusions	The analysis was restricted to spawning observations recorded in the 21st century such that the global atlas of artificial light at night under the sea is broadly representative of probable light exposure during the time of observations. Genera with less than six recorded observations per ALAN exposure level were excluded from the analysis. Data from ecoregions with fewer than six recorded observations were excluded from the analysis. Ex situ observations of coral spawning were omitted from the analysis to remove the influence of nearby light sources from buildings and laboratory light sources. The resulting dataset consisted of 2135 spawning observations recorded at 52 locations in 19 Ecoregions around the world between 2000 and 2019, across 156 species representing 12 genera.
Reproducibility	The analysis was performed using existing publicly available datasets and is entirely reproducible.

Randomization	The analysis was performed on existing long term ecological time series data. While data filters have been applied to the data used in that analysis (outlined above), the authors had no prior influence of the design of the data collection.
Blinding	Data collection was already completed without the tested hypothesis having been conceived and as such the data collection procedures can be considered blind.

Did the study involve field work? ☐ Yes ☒ No

Reporting for specific materials, systems and methods

We require information from authors about some types of materials, experimental systems and methods used in many studies. Here, indicate whether each material, system or method listed is relevant to your study. If you are not sure if a list item applies to your research, read the appropriate section before selecting a response.

Materials & experimental systems

n/a	Involved in the study
☒	☐ Antibodies
☒	☐ Eukaryotic cell lines
☒	☐ Palaeontology and archaeology
☒	☐ Animals and other organisms
☒	☐ Clinical data
☒	☐ Dual use research of concern

Methods

n/a	Involved in the study
☒	☐ ChIP-seq
☒	☐ Flow cytometry
☒	☐ MRI-based neuroimaging
